# Diagnostic Tapping of the Chest

**Published:** 1902-06-14

**Authors:** 


### DIAGNOSTIC TAPPING OF THE CHEST.

Dr. George F. Murray [1] insists on the importance of carefully cleansing the skin with antiseptics, and of thoroughly sterilising the needle, when it is necessary to explore the chest in cases of suspected pleuritic effusion. He says that the best form of needle to use is one made just like a hypodermic syringe, but at least three times as large. It should hold fully a drachm; the hollow needle should be not less than three inches long, and should have an internal diameter of not less than one-sixteenth of an inch. The needle also should be strong and have no flaw in it. Such a needle when fixed on to the syringe held in the hand ought to be able to bear a lateral strain of one pound in any direction near the point. The syringes which are supplied for the injection of antitoxic serum should never be used to explore the chest, as the needle has been known to break off short. For sterilisation Dr. Murray puts the needle into a test tube with enough cold water to cover it. This is then heated over a spirit lamp till it boils, and is kept gently boiling for a minute. The tube is then inverted over a dish containing 1 in 40 carbolic lotion, so that the needle falls directly into this, and is kept sterile till it is wanted. If cultures are to be made from the fluid, both syringe and needle should be boiled in a pan. The disinfection of the skin is efficiently secured by having it well washed with soap and water, and then scrubbed with cotton-wool soaked in 1 in 20 carbolic lotion.

[1] Lancet, May 24.

